# What Is New in the Clinical Management of Low Back Pain: A Narrative Review

**DOI:** 10.7759/cureus.22992

**Published:** 2022-03-09

**Authors:** Enrique Orrillo, Luis Vidal Neira, Fabián Piedimonte, Ricardo Plancarte Sanchez, Smiljan Astudilllo Mihovilovic, Marco Antonio Narvaez Tamayo, Martina Rekatsina, Giustino Varrassi

**Affiliations:** 1 Pain Medicine, Asociacion Peruana para el Estudio del Dolor, Lima, PER; 2 Rheumatology, Centro de Diagnóstico de Osteoporosis y Enfermedades Reumáticas (CEDOR), Lima, PER; 3 Neurosurgery, Fundación CENIT para la Investigación en Neurociencias, Buenos Aires, ARG; 4 Anesthesiology, University Autonomous of Mexico, Mexico City, MEX; 5 Rehabilitation Medicine, Hospital Nacional, Santiago, CHL; 6 Pain Medicine, Hospital Obrero, La Paz, BOL; 7 Pain Management, Basildon University Hospital, London, GBR; 8 Pain Management, Paolo Procacci Foundation, Rome, ITA

**Keywords:** interventional pain medicine, pain medicine, chronic non-specific low-back pain, pain, low-back pain (lbp)

## Abstract

Low back pain (LBP) is a prevalent condition associated with disability. Treating patients with LBP becomes further complicated by the potential presence of underlying conditions, such as cancer or traumatic injury, or biopsychosocial aspects. LBP usually has a neuropathic component that must be assessed and treated appropriately. Pharmacological management of LBP requires a thorough knowledge of the available agents and the mechanisms of the LBP. Although there are effective pharmacological treatments for LBP, it is important to consider safety issues. Fixed-dose combination products may be helpful, as they can reduce opioid consumption without sacrificing analgesic benefits. Neuromodulation is an important and sometimes overlooked treatment option for LBP and may be appropriate for chronic LBP requiring long-term treatment. Imaging studies support neuroplastic changes in the brain as a result of neuromodulation. Interventional approaches to chronic LBP are numerous and must be appropriately selected based on the individual patient. Evidence in support of epidural injections for LBP is strong for short-term pain control but moderate to limited for long-term relief. Rehabilitation for LBP can be an important element of long-term care, and new forms of rehabilitation programs are being developed using telemedicine. A variety of new and established treatments are available for patients with LBP, and clinicians and patients may benefit from emerging new treatment modalities.

## Introduction and background

Low back pain (LBP) is the leading cause of disability worldwide. Despite its near-universality, it is not adequately diagnosed or treated [1]. The lifetime prevalence of LBP may be as high as 84%, and of those who with one episode of LBP, 44%-78% will experience relapse [2]. About a quarter of those who experience LBP will consult with a healthcare provider, of whom 91% seek care specifically from a physician [3,4]. This review is based on presentations from the Lima International Symposium on Pain (https://bit.ly/3Iz59ci), which addressed the care of LBP patients, with an emphasis on the latest research and breakthroughs in the diagnosis, treatment, and rehabilitation of LBP.

## Review

Clinical presentation of LBP

LBP is often described in temporal terms, but it may also be differentiated by etiology and other characteristics (see Table 1 for details). Nonspecific LBP is the most frequently seen type of acute form of the condition, while specific LBP is much less prevalent [5]. Pain management is an important part of treatment as well as a functional improvement [6].

**Table 1:** Clinically helpful ways to describe LBP and associated conditions LBP, low back pain.

Method	Terms	Definitions
Temporal	Acute LBP	0-6 weeks
	Subacute LBP	6-12 weeks
	Chronic LBP	>12 weeks
Etiological	Nonspecific LBP	Cannot be attributed to a known specific pathology
	Specific LBP	Caused by a known pathology, such as radiculopathy or spinal stenosis
Characteristics	Mechanical LBP	Caused by abnormal stress and strain on muscles and soft tissues around the vertebral column
	Inflammatory LBP	Localized LBP in the axial spine and sacroiliac joints, usually occurring with known inflammatory conditions
	Referred LBP	Pain that originates in another location but caused pain in the lower back

**Figure 1:**
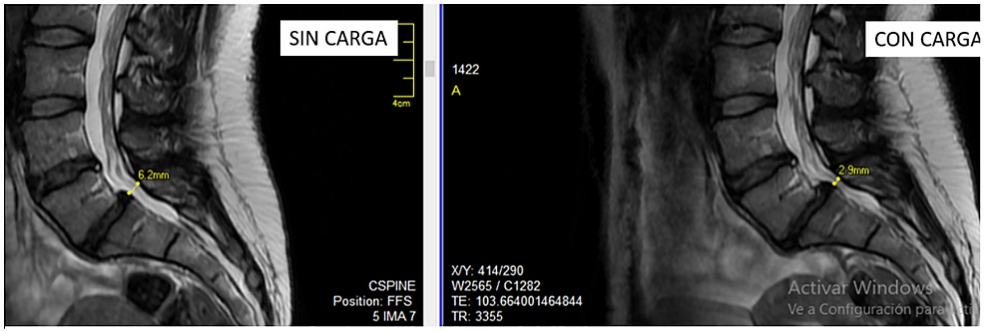
Images showing the same patient with a herniated disc at L5-S1 but the channel diameter is reduced by more than half with a load than without (6.2 mm on left, 2.9 mm on right). Axial loading is not represented when the patients reclined during the MRI MRI, magnetic resonance imaging.

In about 85% of patients, the etiology of LBP remains unknown, even after extensive testing [7]. For adults <50 years with no apparent underlying systemic conditions, imaging may not be necessary, but it may be appropriate for those suspected of having underlying pathology or certainly for those considering surgery [8]. Caution is required with imaging for LBP. Because of axial loading, LBP patients often experience less pain when supine, the position in which they undergo the MRI examination, than they do while walking or standing upright [9]. In a study of 20 patients with spinal stenosis, five patients recommended for conservative treatment by the three neurosurgeons in the study after having viewed their conventional MRI spinal images were reclassified by all three neurosurgeons in the study as appropriate for decompressive surgery after viewing an MRI showing the same patients with the axial load associated with standing [9] (see Figure 1).

The improvement in surgical tools has allowed for the development and expansion of minimally invasive spine surgery such as indirect decompression through the use of devices between the interspinous processes, microscopic spine surgery, and endoscopic procedures [10]. These new techniques offer less injury to tissue, lower rates of complications, and reduced recovery times. It may even be expected that minimally invasive spine surgery will replace conventional open surgery in the near future [10].

Although there is no single definitive “pain center” in the brain, the central nervous system processes and interprets pain through an interplay of complex signaling pathways that are only now beginning to be elucidated. Maladaptive neuroplasticity can result in chronic painful syndromes [11]. An illustrative example occurs in radicular pain, a type of neuropathic pain arising in the spine and radiating outward to the periphery as a result of inflamed or irritated nerve roots. In radicular pain syndromes, the neurons become sensitized to the point that central nervous system sensitization can occur within a matter of hours. Cerebral changes induced by pain, learning, and adaptation play an important but not entirely elucidated role in both acute and chronic pains [12]. In the treatment of lumbar disc herniation, which is responsible for about 5% of all LBP cases but 30% of overall annual costs associated with LBP treatment, MRI scans of spinal structures may not be as helpful in terms of prognosis as brain imaging [13]. In a study of 12 right-handed patients with chronic pain (more than three months) due to lumbar disc herniation and right-sided or left-sided sciatica matched to 12 controls without back pain, spinal MRI and high-resolution brain MRI images were compared [14]. The LBP patients were scheduled for surgery, and in the LBP arm, images were taken one day before surgery and again at four weeks after hospital discharge. Controls were likewise scanned twice about 38 days apart but did not undergo surgery. The spinal MRIs in all of the LBP patients confirmed lumbar disc herniation at L4-5 or L5-S1. All surgeries were successful and normal. Control patients showed no changes in gray matter in the brain, but among the LBP patients, there was reduced gray matter volume in the left hippocampus following lumbar discectomy. The scans also revealed a postoperative increase in gray matter volume in the right pallidum and putamen. These changes were not to be associated with variations in pain intensity levels [14]. Chronic LBP is associated with decreased cortical thickness in various areas of the brain, and effective pain control appears to reverse these changes by increasing cortical thickness [15]. Increased cortical thickness in the left dorsolateral prefrontal cortex could be correlated to reductions in pain, dysfunction, and cognitive impairment. This supports the promising concept that pain-related neuroanatomical changes may be reversed with effective pain care [15].

Pharmacological therapy

The pharmacological treatment of LBP must consider multiple factors as described in Table 2 and may utilize any number of agents alone or in combination. A Delphi study on optimal treatment of LBP favored multimodal therapy and a multidisciplinary approach [6]. Based on a meta-analysis, the main drug classes described in Table 3 may be effective in treating LBP [16].

**Table 2:** Chart for patients presenting with LBP, assuming the clinician has physically examined the patient and taken a detailed patient history LBP, low back pain.

Patient findings	Possible diagnosis	Recommendations
The patient has pain only in the lower back and has some functional limitations	Nonspecific LBP	No further testing or examination is needed. General symptomatic treatment
There is suspicion or determination of a specific underlying pathophysiological cause and/or “red flags”	Specific LBP	Further examination and testing are needed to confirm the specific underlying cause. Treatment is based on the specific cause of the pain
Neurological pain symptoms or signs of radiculopathy are present or suspected	Neurological syndrome or radicular LBP	Further examination and testing are needed to determine the cause of the pain. Consultation with a neurologist may be appropriate

**Table 3:** Based on randomized, placebo-controlled clinical trials, a meta-analysis reported on the main classes of agents that may be used in treating [16]. The evidence for their utility in acute and chronic LBP reveals that there is no ideal first-line treatment for LBP NSAIDs, nonsteroidal anti-inflammatory drugs; SSRIs, selective serotonin reuptake inhibitors; LBP, low back pain.

Drug	Acute		Chronic		Comment
	Pain	Function	Pain	Function	
Acetaminophen	No effect	No effect	No effect	No effect	
NSAID	Slight effect	Slight effect	Slight to moderate effect	Slight to no effect	Low-quality evidence. Safety concerns with long-term use of NSAIDs
Opioids	No evidence	No evidence	Slight effect	Slight effect	Patches are less effective than strong opioids
Muscle relaxants	Effective	No effect	Negligible effect	Not reported	Not recommended for long-term use
Benzodiazepines	Negligible	No evidence	No benefit	Not applicable	Not recommended for long-term use
Anticonvulsants	No evidence	Negligible effect	Negligible effect	Not evident	
Systemic corticosteroids	No effect	No effect	Not applicable	Not applicable	
Tricyclic antidepressants	Not applicable	Not applicable	No effect	No effect	
SSRIs	Not applicable	Not applicable	No effect	Not applicable	
Duloxetine	Not applicable	Not applicable	Slight effect	Slight effect	Moderate quality evidence
Tramadol	Not applicable	Not applicable	Moderate	Slight	Moderate quality evidence

Despite the fact that acetaminophen (paracetamol) is often recommended as a first-line treatment for acute LBP, by-the-clock or as-needed use of acetaminophen was not more effective than placebo in reducing pain intensity or shortening recovery time [17]. Furthermore, acetaminophen is associated with cardiovascular risk, which may limit its long-term use. In a study of incident hypertension in two cohorts of older (51-77 years) and younger (34-53 years) women, the consumption of acetaminophen, nonsteroidal anti-inflammatory drugs (NSAIDs), and aspirin was compared for incident hypertension. Patients who took >500 mg/day acetaminophen had a multivariable relative risk of 1.78 (older) and 1.60 (younger) for incident hypertension, and the risk was dose-dependent. Higher daily consumption of NSAIDs also had a similar effect, but aspirin did not [18]. A systematic review of eight observational studies comparing the use and non-use of acetaminophen reported cardiovascular adverse events (*n*=4) and found a dose-dependent increased risk ratio of cardiovascular adverse events ranging from 1.19 to 1.68 [19].

In a systematic review and meta-analysis of the use of NSAIDs to treat acute LBP (*n*=32 trials, 5,356 patients), moderate-quality evidence showed that NSAIDs reduced pain intensity more than placebo and there was high-quality evidence that they were more effective than placebo in the improvement of short-term disability [20]. However, NSAIDs are associated with adverse events of gastrointestinal, cardiovascular, cutaneous, renal, hematological, respiratory, and central nervous systems [21-23]. Nonselective NSAIDs increase the risk of peptic disease by a factor of five. They increase the risk of upper gastrointestinal bleeding four-fold. Selective cyclooxygenase-2 (COX-2) inhibitors have lower rates of gastrointestinal toxicity [24] but are associated with cardiovascular risk [25]. When nonselective NSAIDs are used, a proton-pump inhibitor may be co-prescribed to help mitigate gastrointestinal risks.

Muscle relaxants can be effective for short-term use in the treatment of acute LBP but their use is controversial because they are associated with adverse events that can be treatment-limiting [26,27]. A brief overview of the main muscle relaxants appears in Table 4. Before prescribing a muscle relaxant, it may be helpful to determine if and how frequently muscle spasms are occurring and if there are signs of Forestier’s disease, diffuse idiopathic skeletal hyperostosis, where the soft tissues such as ligaments and tendons thicken and calcify [28]. 

**Table 4:** Summary of adverse events associated with the main muscle relaxants used to treat acute LBP LBP, low back pain.

Agent	Dose	Adverse events
Carisoprodol	350 mg/6 h	Dizziness, somnolence, headache, allergic reactions, and idiosyncratic reactions (mental status change, quadriplegia, temporary loss of vision)
Clorzoxazone	250-750 mg/6-8 h	Dizziness, somnolence, red urine, gastrointestinal irritation, gastrointestinal bleeding (rare), and hepatotoxicity. Severe allergic reactions are possible
Cyclobenzaprine	5-10 mg/8 h	Anticholinergic effects (dizziness, somnolence, and increased intraocular pressure), rare but serious side effects include arrhythmias, convulsions, and acute myocardial infarction
Diazepam	2-10 mg/6-8 h	Dizziness, somnolence, confusion, and abuse potential
Metaxalone	800 mg/6-8 h	Dizziness, somnolence, headache, nervousness, leukopenia or hemolytic anemia (rare), hepatotoxicity, and muscle spasms
Metacarbamol	750-1500 mg/6 h	Dark urine, change in mental status, and worsening of myasthenia gravis
Orphenadrine	100 mg/12 h	Anticholinergic effects (dizziness, somnolence, and increased intraocular pressure), aplastic anemia (rare), gastrointestinal irritation, and allergic reactions
Tizanidine	2-4 mg/6-8 h	Dose-related hypotension, sedation, dry mouth, hepatotoxicity, and rebound hypertension upon discontinuation

In a systematic review and meta-analysis of 31 trials (*n*=6,505 acute LBP patients), evidence for muscle relaxants was equivocal. Low-quality evidence found that non-benzodiazepine antispasmodic agents reduced pain intensity in the first two weeks but did not improve function. Similarly, there was low-quality evidence that a non-benzodiazepine muscle relaxant might increase the risk of an adverse event [29]. In a randomized, double-blind study of emergency department patients presenting with acute LBP treated with naproxen (500 mg/twice daily) plus diazepam versus naproxen plus placebo, the combination of naproxen plus diazepam did not significantly reduce pain or improve function at one week and three months post-discharge compared to naproxen alone [30]. Benzodiazepines are not recommended for use in patients with acute lumbar disc prolapse [31,32]. Caution should be used when prescribed benzodiazepines for more than short-term use as indicated [33].

Pharmacological management for chronic LBP must be suitable for long-term treatment. In a systematic review of 15 clinical trials (*n*=5,540) of LBP patients, opioids were shown to be effective in the short term for reducing pain and somewhat effective for improving function compared to placebo in chronic LBP patients [34]. However, opioid-associated side effects can be distressing to some patients. Frequently reported opioid-associated side effects include nausea (8%), dizziness (8%), constipation (7%), vomiting (7%), somnolence (6%), dry mouth (6%), and others (<5%) including headache, pruritus, fatigue, anorexia, and hyperhidrosis [34].

Fixed-dose combination products combine two agents with complementary mechanisms of action into a single pill or capsule. Many types of combination therapies have been shown to reduce opioid consumption without sacrificing analgesic benefits [35]. Examples of fixed-dose combination products include acetaminophen/oxycodone, dexketoprofen/tramadol, acetaminophen/hydrocodone, and diclofenac/thiocolchicoside. A study of 82 acute LBP outpatients compared the efficacy and tolerability of dexketoprofen/tramadol 75/25 mg to diclofenac/tiocolchisoide 75/4 mg [36]. In this study, patients had acute LBP caused by a herniated disc rated above four on a zero-ten pain scale. Patients received oral dexketoprofen/tramadol or intramuscular injection of diclofenac/tiocolchisoide; treatments were administered once every 12 h over a five-day course. Dexketoprofen/tramadol 75/25 mg provided significantly superior and more sustained analgesia at days three and seven and had a higher proportion of respondents at days one, three, and seven (75.0% vs. 71.1%; 93.2% vs. 73.7%; and 95.5% and 71.1%, respectively). The dexketoprofen/tramadol 75/25 mg group also exhibited a significantly greater reduction in neuropathic pain. Both treatments were similarly well tolerated [36].

Antidepressants are increasingly considered for analgesic benefits. A systematic review evaluated 23 randomized clinical trials using antidepressants to treat LBP, and, compared to placebo, antidepressants decreased pain intensity by 4.3 points on a 0-100 scale. However, the use of antidepressants in LBP was associated with a significant risk for stopping treatment for any reason (odds ratio 1.27). The reduction in pain for these patients was deemed clinically unimportant, while the use of antidepressants exposed patients to the risk of antidepressant-associated side effects [37].

Anticonvulsants such as gabapentin, pregabalin, and topiramate are sometimes used to treat LBP, but a systematic review of nine studies (*n*=859) found moderate- to high-quality evidence that these agents were not effective in treating either LBP or lumbar radicular pain. Gabapentinoids (gabapentin and pregabalin) expose patients to the risk for adverse effects, of which the most frequently reported were somnolence, dizziness, and nausea [38]. However, in real-world clinical practice, the use of anticonvulsants can sometimes be helpful because they address the neuropathic component of some forms of LBP. Using pooled data from companion eight-week prospective, observational studies of chronic LBP, 700 patients were treated with pregabalin either as monotherapy or in combination with other analgesics versus usual care (NSAIDs but no pregabalin). All outcomes were significantly improved in the pregabalin group compared to the control group, and over 50% of the pregabalin patients could be counted responders (≥50% pain reduction) compared to less than 50% in the control group. However, 36.1% of the pregabalin patients experienced adverse events. The most commonly reported adverse events for the pregabalin patients were dizziness (10.3%) and somnolence (8.9%) and 7.0% of pregabalin patients discontinued treatment because of side effects [39].

In developed nations, LBP patients typically receive pharmacological treatment [40]. A systematic review of European clinical practice guidelines for neck and LBP provides consensus recommendations, as summarized in Table 5 [41].

**Table 5:** Synthesis of 17 European guidelines in eight European countries for pharmacological treatments for neck pain and LBP LBP, low back pain; NSAIDs, nonsteroidal anti-inflammatory drugs. Source: [41].

Agent	Guidelines	Countries	Recommendation	Strength of evidence
Acetaminophen	8	6	Against	Moderate
NSAIDs	9	7	Equivocal	
Opioids (including tramadol) combined with acetaminophen or NSAIDs	8	6	Equivocal	
Antidepressants	6	5	Against	Strong
Anticonvulsants	6	6	Against	Strong
Muscle relaxants	5	5	Against, with some exceptions	Strong
Topical agents, including topical NSAIDs	3	3	Inconclusive	

Neuropathic component of LBP

Neuropathic pain is distinguished by the fact that there is no transduction of a nociceptive signal into an electrical impulse and there may be an injury to major nerves. In contrast to other forms of pain, neuropathic pain tends to be less responsive to conventional analgesic therapy [42]. LBP often has a neuropathic component, as was shown when the painDETECT questionnaire was utilized in an unselected cohort of approximately 8,000 chronic LBP patients in Germany, which then determined that about 37% of these LBP patients had mainly neuropathic pain. This subpopulation with a neuropathic component to their LBP tended to have greater pain intensity, more numerous and more severe comorbid conditions, such as depression and anxiety, and a higher rate of sleep disorders [43]. Extrapolated from this data, it was determined that about 14.5% of all female and 11.4% of all male chronic LBP patients in Germany have some degree of neuropathic pain [43]. A meta-analysis of 20 studies (*n*=14,269 LBP patients) used a pooled analysis and reported that 55.8% of LBP patients had a neuropathic component to their pain, and neuropathic pain was more likely to occur in those LBP patients with concomitant leg pain than in those with uncomplicated LBP [44].

The neuropathic component of LBP can be assessed using the Douleur Neuropathique 4 (DN4) questionnaire, which has been shown to possess 83% sensitivity and 90% specificity, along with a positive predictive value of 86% [45] A Spanish version of this assessment tool, the first translation of the survey, has been validated in a study of 164 patients [46]. The survey consists of 10 items, seven questions for the patient about pain characteristics and three items based on the clinician’s examination of patients, such as the presence or absence of tactile allodynia. Patients who score above four are positive for neuropathic pain.

Interventional procedures and neuromodulation

Epidural corticosteroids have been used for decades to treat lumbar stenosis [47] as well as radiculopathy [48]. Steroids decrease the production of inflammatory mediators in the nerve roots and reduce the concentrations of endogenous pro-inflammatory cytokines [48]. In a systematic review and meta-analysis of lumbar spinal stenosis patients with neurogenic claudication, there was only low-quality evidence that found epidural steroids reduced pain, improved function, or enhanced quality of life at two weeks compared to home exercise or inpatient physical therapy [49]. Another systematic review and meta-analysis reported that epidural corticosteroid injections slightly reduced leg pain and disability in patients with lumbosacral radicular pain with minimal and minor adverse events [50]. The American Society of Interventional Pain Physicians (ASIPP) issued guidelines for epidural injections based on the nature of the back pain and the type of injection. It found strong evidence favoring fluoroscopically guided injections for lumbar stenosis and moderate evidence supporting lumbar transforaminal epidural injections for long-term improvements [51]; however, the effectiveness of these treatments is limited [52-57] (see Table 6). 

**Table 6:** Quality of evidence regarding epidural steroid injections for treatment of LBP. Appropriate patient selection leads to better optimized results LBP, low back pain.

	Quality of evidence for pain control		Comments
	Long term	Short term	
Caudal epidural block	Moderate	Strong	Discal hernia, radiculitis, and discogenic pain
Interlaminar epidural block	Limited	Strong	
Selective nerve block	Moderate	Moderate	May allow surgery to be delayed, second-line approach
Transforaminal block	Limited	Strong	Chronic LBP and pain in lower extremities

Endoscopic rhizotomy for denervation of the lumbar facet joins was studied in 50 consecutive LBP patients treated in the emergency department of a single center. Patients were followed up for two years, and it was found that endoscopic rhizotomy was effective at two years for reducing facet joint pain. Since 20%-40% of LBP patients have some degree of facet joint inflammation, this may be an important procedure to consider [58].

Neuromodulation is available using different technologies, systems, and devices, such as transcutaneous nerve stimulation, peripheral nerve stimulation, dorsal root ganglion (DRG) stimulation, deep brain stimulation, as well as the use of intraspinal and/or intracerebroventricular agents. Delivered by electrodes implanted percutaneously or by laminectomy into the epidural space adjacent to the spinal cord, neuromodulation may produce sympatholytic effects [59]. Neuromodulation is a less-invasive and reversible treatment compared to open surgery or other interventions, offering a good alternative for chronic pain control in certain lumbar stenosis patients [60]. Based on a systematic review of 161 randomized clinical trials, the ASIPP recommends that spinal cord stimulation be discussed as a treatment option for patients with persistent and/or disabling radicular pain, but with disclosure of the fact that this treatment may be associated with postoperative complications [61].

In a study of 50 patients who underwent surgery to treat persistent or recurrent radicular LBP, patients who still had pain were randomized postoperatively to receive either spinal cord stimulation or reoperation. Spinal cord stimulation was significantly more successful than surgery (*p*<0.01), and spinal cord stimulation patients consumed significantly less opioid analgesics than surgical patients. Pain relief >50% was achieved in 38% of spinal cord stimulation patients compared to 12% of reoperation patients at a mean follow up of 2.9 years [62]. In a study of 100 patients with failed back surgery syndrome, patients were randomized to receive either conventional therapy alone or conventional therapy plus spinal cord stimulation. At six months, 48% of spinal cord stimulation and 9% of controls achieved >50% pain relief in the legs. The spinal cord stimulation group had significantly greater back pain relief, improved quality of life, and better function than the controls. At 12 months, 32% of device patients had at least one device-related complication [63]. Such complication rates are not unusual and most commonly involve electrode migration, infection, or complications around the generator pocket [63]. 

Implantable targeted drug delivery systems, sometimes called intrathecal drug pumps, are used to deliver a small amount of medication into the intrathecal space around the spine [53]. They are currently indicated for spasticity (targeted baclofen delivery) and refractory pain [64]. Targeted drug delivery systems are supported by strong evidence for the short-term relief of neuropathic and/or cancer pain and moderate evidence for the long-term relief of chronic pain [64]. Neuromodulation may be indicated for patients who have chronic LBP and who fail to respond to conservative therapies, have a positive electromyography test, or who continue to have pain after back surgery. Any LBP patient not specifically contraindicated for neuromodulation may be considered for this sort of treatment [65].

Focal stimulation of the DRG appears to provide greater pain relief for complex regional pain syndrome than conventional spinal cord stimulation. In a study of patients with either LBP or pain in the lower limbs, overall pain levels were reduced using DRG stimulation by a mean of 56% at 12 months after device implant, and patients reported a high level of satisfaction with the therapy [66]. In a study of 12 patients with chronic discogenic LBP caused by failed back surgery syndrome, more than half of the patients reported ≥50% pain relief at 12 months, were able to reduce analgesic consumption, and had a better quality of life. Average LBP relief was 45.5% at one year [67].

Continuous radiofrequency (RF) lesioning adjacent to the DRG has been effective in treating radicular pain [68], and pulsed RF lesioning has been used in treating lumbosacral radicular pain [69]. A retrospective review of patients treated with pulsed or continuous RF lesioning of the lumbar DRG and segmental nerve were identified in medical records and 40 cases were revealed where the patient had ≥50% pain relief after the procedure to treat either lumbar DRG or sacral segmental nerve pain [70]. The mean age of the patient was 62 years (25-86 years), and the mean duration of relief for those who had two treatments was 4.7 months (0-24 months). The mean duration of relief and success remained constant after each subsequent RF treatment. One adverse event was reported for transient sensations of numbness in the thigh, which resolved spontaneously after one week [70]. While there are decades of experience with both pulsed and continuous RF applications, their mechanisms of action remain to be elucidated [71,72]. A comparison of the two RF approaches appears in Table 7.

**Table 7:** Comparison between continuous and pulsed RF therapeutic approaches to control LBP RF, radiofrequency; LBP, low back pain.

	Continuous RF	Pulsed RF
First use	1975	1998
Application	Continuous RF energy for 90 s	RF energy in 20-ms pulses with a washout period of 480 ms
Needle tip	Parallel and by side of the target	Perpendicular, pointing at the target
Tissue temperature	Up to 80^o^C	Up to 42^o^C
Proposed mechanism of action	Nonselective thermal destruction	Neurobiological, using strong electrical fields
Side effects	Deafferentation syndrome	None observed
Duration of effect	Potentially months	Shorter duration than continuous RF
Use on peripheral nerves	No, contraindicated	Yes, has been successfully used in peripheral monotherapies

Overall, LBP treatment seems to be migrating away from open surgery toward more minimally invasive procedures, neuromodulation, and other therapies. Neuromodulation is relatively new but various treatment options have already demonstrated positive and promising results in terms of effectiveness with few side effects.

Clinical challenges

Treating LBP can be challenging due to the diversity of presentations, different causes and exacerbating factors, as well as wide interpatient variability in mental health status, age, comorbidities, lifestyle choices, genetic factors, socioeconomic status, and underlying conditions, all of which can play a role in back pain symptoms [73]. However, our increased understanding of the complexities of LBP and its mechanisms provides an impetus for improved diagnostic procedures, evidence-based treatments, and the development of more precisely targeted interventions. Managing chronic LBP may require a multidisciplinary clinical team and a willingness to explore and integrate psychological and social aspects as well as anatomical and biological factors into patient care [73]. Holistic care, shared decision-making, and individualized treatments are important considerations [74]. Drug therapy may require a combination of agents and multimodal approaches [75].

The term “intractable LBP” may actually be a misnomer, because viewing LBP as a multi-mechanistic condition requires a multimodal approach, and hence an effective treatment may be possible. The appropriate treatment depends, in part, on accurate pain classification and understanding of the mechanisms of pain; combination treatment, such as pharmacological and nonpharmacological methods, may be required to provide relief [76]. Combination therapy may go beyond pharmacological agents alone and combine interventional treatment, lifestyle changes, injections, psychological counseling, exercise, weight loss, drug therapy, and so on [77]. This may necessitate a multidisciplinary clinical team and referrals to pain specialists.

When treating intractable LBP, clinicians must recalibrate their goals: reducing or at least managing the pain while restoring functions as much as possible. This involves providing behavioral and psychological support to maintain the patient’s progress. When caring for patients with intractable LBP, it may be crucial to manage patient expectations, because complete pain relief may never be possible. Among the many treatment options for intractable LBP are percutaneous interventions, which should be selected based on the pain mechanism [77] (see Table 8).

**Table 8:** Interventional percutaneous procedures for treating chronic LBP, including intractable chronic LBP LBP, low back pain; RF, radiofrequency.

Pain source	Treatments
Facet pain	Intra-articular injections, medial branch blocks, and facet neurotomy (RF, cryoablation, neurolysis)
Lumbar stenosis	Implantable devices, RF ablation, transforaminal block, epidural block (interlaminar, caudal), and selective nerve root blocks
Discogenic disease	DiscTrode, annuloplasty, biacuplasty, percutaneous discectomy, ozone therapy, nucleoplasty, hydrodisectomy, delompressor (Stryker, Kalamazoo, Michigan), percutaneous lumbar disc decompression
Rami communicans	Rami communicans nerve blocks
Other lumbar pathologies	Percutaneous or endoscopic lumbar adhesiolysis and epiduroscopy
Sacroiliac joint pathology	Intra-articular injections and RF neurotomy
Additional options	Regenerative medicine, platelets-rich plasma

Future directions

Regenerative medicine uses autologous or allogenic biologics to help the body repair itself by replacing or restoring damaged tissue. As a relatively new medical subspecialty, it must be viewed with professional caution, although regenerative approaches seem to hold promise. There is limited evidence in support of regenerative therapies for the treatment of certain types of LBP [78].

Despite great advances in medical knowledge and remarkable breakthroughs in technology and drug development, there remain important challenges and knowledge gaps in the treatment of LBP. Despite greater concordance among international guidelines on LBP, medical science has had only limited success in identifying safe, effective treatments. In real-world clinical practice, LBP patients typically present with multifactorial pathologies and comorbidities that often require complex and highly individualized treatments. Such advanced and nuanced care is not always provided.

Chronic pain must be considered a biopsychosocial phenomenon rather than just a sensory one, so it is appropriate to ask LBP patients about their lifestyle, social situation, employment, current stressors, diet, use of alcohol and other substances, family situation, and health habits [79]. By reconceptualizing pain and discussing it in the context of the patient’s broader life, the patient may become more empowered during the rehabilitation process. Of course, the pandemic drastically changed rehabilitation. During the lockdown period, telerehabilitation came into use [80]. This may be an important step in helping patients manage the difficult path of self-guided rehabilitation efforts. In addition to online and device-based applications intended to guide rehabilitation efforts, some devices that can facilitate specific exercises and postures. Other software applications (apps on smartphones, for example) may be able to connect the patient with the clinic or a clinical service to monitor progress or answer questions.

LBP remains a global health challenge but our growing appreciation of its complex etiology, multimodal drug therapy, interventional procedures, and new advances may facilitate future treatment and restore function and comfort to the many LBP patients seeking care.

## Conclusions

LBP remains a serious, prevalent, and challenging global public health problem that requires a multidisciplinary clinical solution. Much chronic LBP has a neuropathic component, which may require a multimodal analgesic approach. Numerous treatment options exist for LBP which often must work in combination with each other: pharmacological therapy, physical rehabilitation, lifestyle changes, neuromodulation, interventional approaches, surgery, and psychological support. Since LBP is not a monolithic condition, treatment must be individualized for each patient, and the choice of optimal therapy and rehabilitation depends on the etiology of the LBP as well as patient factors.
